# Engineering chimeric antigen receptor T cells for solid tumour therapy

**DOI:** 10.1002/ctm2.1141

**Published:** 2022-12-10

**Authors:** Longwei Liu, Yunjia Qu, Leonardo Cheng, Chi Woo Yoon, Peixiang He, Abdula Monther, Tianze Guo, Sarah Chittle, Yingxiao Wang

**Affiliations:** ^1^ Department of Bioengineering Institute of Engineering in Medicine University of California La Jolla California USA

## Abstract

Cell‐based immunotherapy, for example, chimeric antigen receptor T (CAR‐T) cell immunotherapy, has revolutionized cancer treatment, particularly for blood cancers. However, factors such as insufficient T cell tracking, tumour heterogeneity, inhibitory tumour microenvironment (TME) and T cell exhaustion limit the broad application of CAR‐based immunotherapy for solid tumours. In particular, the TME is a complex and evolving entity, which is composed of cells of different types (e.g., cancer cells, immune cells and stromal cells), vasculature, soluble factors and extracellular matrix (ECM), with each component playing a critical role in CAR‐T immunotherapy. Thus, developing approaches to mitigate the inhibitory TME factors is critical for future success in applying CAR‐T cells for solid tumour treatment. Accordingly, understanding the bilateral interaction of CAR‐T cells with the TME is in pressing need to pave the way for more efficient therapeutics. In the following review, we will discuss TME‐associated aspects with an emphasis on T cell trafficking, ECM barriers, abnormal vasculature, solid tumour heterogenicity and immune suppressive microenvironment. We will then summarize current engineering strategies to overcome the challenges posed by the TME‐associated factors. Lastly, the future directions for engineering efficient CAR‐T cells for solid tumour therapy will be discussed.

## Graphical abstract

The tumour microenvironment (TME) is a complex and evolving entity composed of different cell types, vasculature, soluble factors and extracellular matrix, with each component playing a critical role in affecting chimeric antigen receptor T (CAR‐T) cell immunotherapy. In this review, we have summarized current CAR‐T engineering approaches developed to overcome such challenges posed by the TME‐associated factors and discussed future directions.

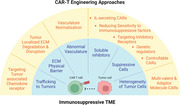

## 1 INTRODUCTION

**FIGURE 1:**
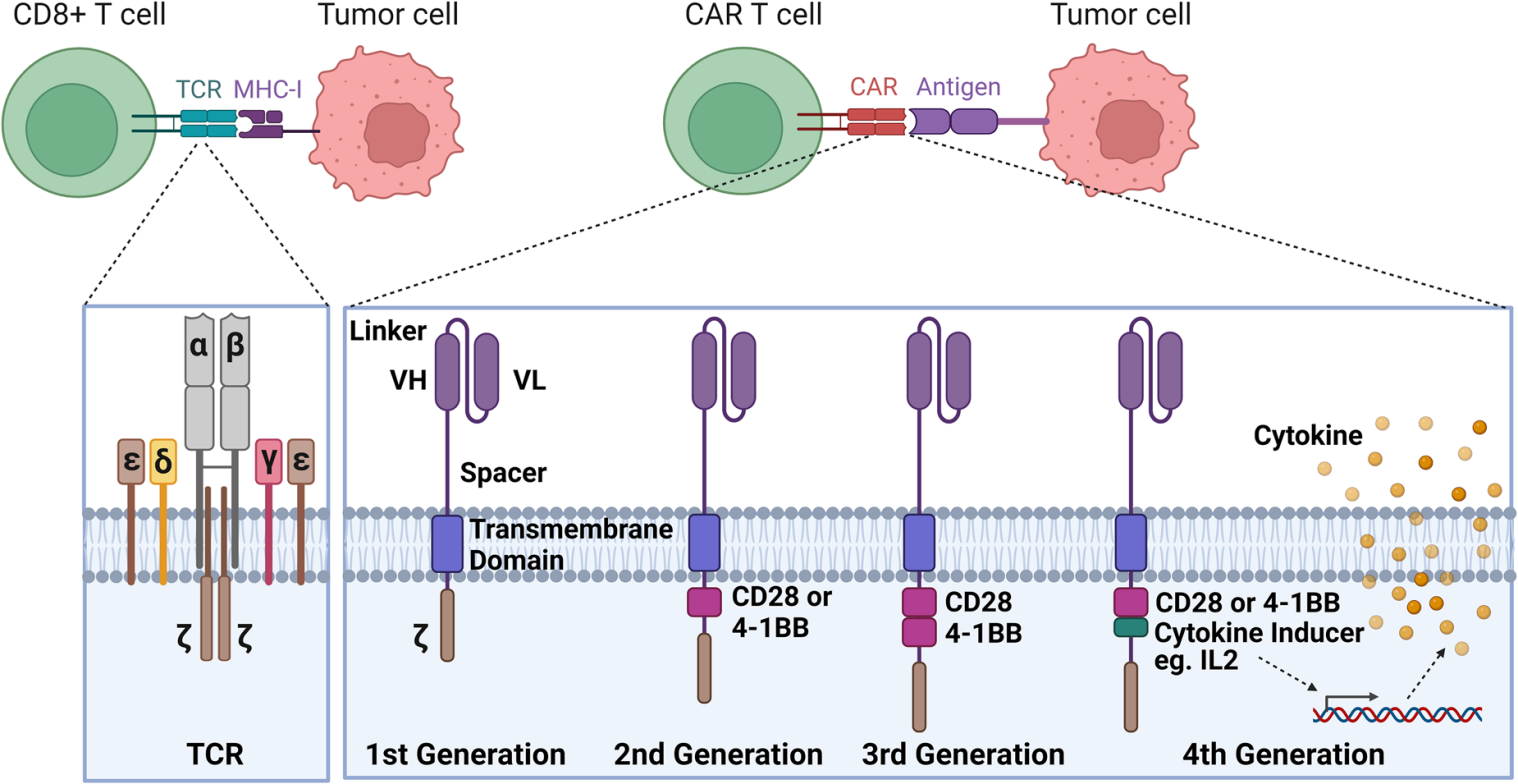
T‐cell receptor (TCR) versus engineered chimeric antigen receptor (CAR) T cells. TCR‐MHC molecule interaction is involved in CD8+ T cell killing of the tumour cells. CAR proteins are engineered in symmetry to redirect T cells for targeting specific antigens on the tumour cells. CARs are composed of an extracellular portion (VH, variable heavy chain; VL, variable light chain.), a transmembrane domain and a CD3ζ region. Second‐generation CAR also includes a co‐stimulatory endodomain fused to CD3ζ (CD28 or 4‐1BB); third‐generation CAR contains two co‐stimulatory domains; transgenic cytokine expression is induced in the tumour tissue upon CAR signalling activation

**FIGURE 2:**
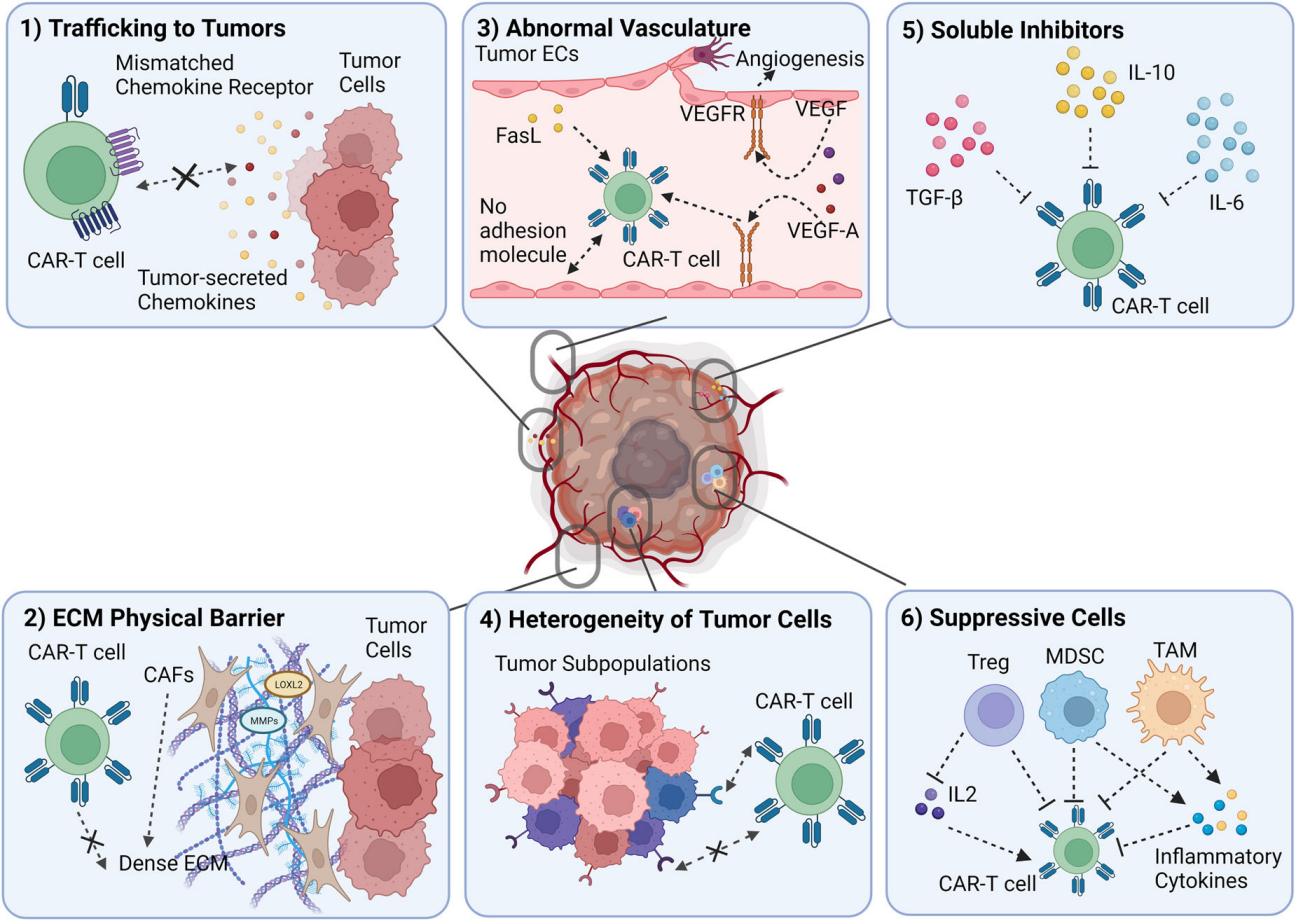
A schematic overview of the immunosuppressive tumour microenvironment for chimeric antigen receptor (CAR) T cells. CAR‐T cells efficacy is hindered in the overall immunosuppressive tumour microenvironment (TME) due to^1^ trafficking to the tumour site,^2^ extracellular matrix (ECM) which serves as a physical barrier,^3^ the abnormal vasculature and angiogenesis,^4^ the heterogeneity of tumour cells which impedes CAR‐T in antigen recognition,^5^ soluble suppressive inhibitors which inhibit T cell function and^6^ other suppressive cell types

Chimeric antigen receptors (CARs) are recombinant receptors that are designed based on the natural T‐cell receptor (TCR) and when expressed on T cells, they can redirect the T cells to recognize and target tumour‐specific surface antigens^1^, ^2^ (Figure 1). CARs are composed of an extracellular antigen‐recognition domain, which typically is a single‐chain variable fragment (scFv) antibody, connected by a spacer or hinge and a transmembrane domain to a cytoplasmic signalling domain, where a costimulatory fragment can be included to trigger T cell activation.^3^ CAR‐T cell therapy is becoming a paradigm‐shifting therapeutic approach for cancer treatment, especially with the production of derived memory T cells that can last for years to suppress relapse.^4^, ^5^ Largely due to the success of CAR‐T therapies against blood cancers in clinical studies, the US Food and Drug Administration (FDA) has recently approved several CAR‐T products and therapies targeting CD19 or B‐cell maturation antigen that can be used to treat B cell acute lymphoblastic leukemia (ALL), non‐Hodgkin lymphoma and multiple myeloma.^6^, ^7^, ^8^, ^9^, ^10^ However, despite the striking successes of CAR‐expressing T cells in treating hematological malignancies, CAR‐T‐based therapy has limited success for solid tumours. Currently in the market, all of the six FDA‐approved CAR‐T cell therapies (Abecma, Breyanzi, Carvykti, Kymriah,Tecartus, and Yescarta) were designed for hematological malignancies. Most CAR‐T therapies targeting solid tumours remain in Phase I/II clinical trials and pre‐clinical studies. Many challenges stem from the features of solid TME factors compared to hematological malignancies (Figure 2). In the following sections, we will first introduce the current challenges in CAR‐T cell‐based therapy and then discuss the engineering approaches to address them.

### 1.1 Increasing trafficking of CAR‐T cells to solid tumours

#### 1.1.1 Insufficient trafficking of CAR‐T cells to solid tumours

Adoptive T cell immunotherapy (ACT), especially CAR‐T cell‐based immunotherapy, is a powerful treatment for tumour regression. However, ACTs are far from being used as a first‐line treatment for cancers in clinical practice as they still need to be optimized for efficient T‐cell tracking and anti‐tumour efficacy.^11^, ^12^ In the TME, chemokines are mediators that regulate cell migration and homing of immune cells,^13^ and are secreted by tumour cells, stromal cells, or immune cells.^14^ Thus, one promising strategy to address this inadequacy is arming T cells with tumour‐specific chemokine receptors to improve homing and antitumor activity.

#### 1.1.2 Navigating the CAR‐T cells into the solid tumours

An early study to arm T cells with tumour‐specific chemokine receptors was done to target Hodgkin tumour cells, for which T cells were engineered to produce high thymus‐ and activation‐regulated chemokine/CC chemokine ligand 17 (TARC/CCL17) and macrophage‐derived chemokine.^15^ Antonio et al. engineered CD30 CAR‐T cells to express a receptor recognizing the chemokines (CCR4) and showed improved migration towards a gradient of chemokine ligand TARC and enhanced anti‐lymphoma effects in vivo.^15^ Similarly, chemokine receptor CCR2b was engineered to co‐express with CAR molecules to enhance the trafficking of CAR‐T cells towards tumours.^16^, ^17^ Moon et al. identified that chemokine CCL2 was abundantly expressed in malignant pleural mesothelioma (MPM) tumours while its corresponding receptor CCR2 was poorly expressed in T cells.^16^ This finding led to the engineered expression of CCR2b in CAR‐T cells targeting mesothelin (mesoCAR+CCR2b T cells), resulting in improved tumour killing both in vitro and in vivo. Furthermore, a large abundance of mesoCAR+CCR2b T cells were found in the tumours, revealing enhanced T cell trafficking and infiltration.

More recent studies have demonstrated that highly efficient trafficking and tumour control can be achieved by introducing chemokine receptors to T cells which match the chemokines secreted by target tumour cells. For example, Whilding et al. engineered interleukin 8 (IL‐8) receptors CXCR1 or CXCR2 in αvβ6‐targeted CAR T‐cells and demonstrated the superior anti‐tumour activity of these armed CAR‐T cells against established αvβ6‐expressing ovarian or pancreatic tumour xenografts.^18^ Jin et al. found enhanced migration and antitumor efficacy from the CD70 CAR‐T cells expressing CXCR1 and CXCR2 targeting glioblastoma (GBM), ovarian and pancreatic tumours that secreted IL‐8 (also known as C‐X‐C motif chemokine ligand 8 [CXCL8]) or had radiation‐induced IL‐8.^19^ These modified CD70 CARs expressing CXCR1 (CAR‐R1) or CXCR2 (CAR‐R2) showed more efficient tumour trafficking than the control CD70 CAR only groups after therapy, and the tumour shrinkage was also observed in CAR‐R1 and CAR‐R2 groups while the control CAR and vector‐transduced groups failed to control the tumour growth in mice. A similar approach was used for the treatment of hepatocellular carcinoma. Liu et al.^20^ used Glypican‐3 (GPC3) CAR‐T cells expressing CXCR2 (CXCR2 CAR‐T) to treat hepatocellular carcinoma which showed relatively high expressions of several CXCR2 ligands. Although it was found that CXCR2 expression had no influence on the cytotoxicity of CAR‐T cells in vitro, CXCR2 CAR‐T cells did enhance the infiltration and expansion in the tumour site in vivo with improved trafficking and anti‐tumour responses. In a recent study, Lesch et al. found that CXCL16 is most secreted in murine pancreatic cancer, but its corresponding receptor, CXCR6, was minimally expressed in circulating T cells.^21^ Therefore, T cells overexpressing CXCR6 were compared to other chemokine receptors, CXCR3 and CCR4. Results demonstrated that CXCR6 had more effective anti‐tumour responses with prolonged tumour rejection occurring in 33.3% of mice, whereas mice treated with CXCR3 or CCR4 had only 16.7%–0% prolonged tumour rejection. Furthermore, it was found that CXCR6‐transduced T cell infiltration had more mobility in the tumour tissue in vivo when compared to control T cells without CXCR6. In summary, these findings demonstrated that chemokine receptors corresponding to chemokines enriched at tumour sites can be utilized to increase T cell recruitment and trafficking to the tumours.

### 1.2 Engineering CAR‐T cells to target the aberrant vasculature

#### 1.2.1 Aberrant vasculature in TME and its influence on CAR‐T cells

Tumour vasculature is the first obstacle that CAR‐T cells must overcome after trafficking to the tumour site and before engaging with tumour cells. However, aberrant vasculature often forms in a solid tumour, which can hinder T cell infiltration and develop immunosuppressive tumour microenvironment.^22^, ^23^ A key feature of tumour vasculature is to induce adhesion dysfunction in T cells, mainly in the form of reduced expression of ligands required for T cell adhesion and extravasation, including intercellular adhesion molecule‐1 (ICAM‐1),^24^ ICAM‐2 and vascular cell adhesion molecular‐1.^25^ In addition, blood vessels in the TME are often structurally and topologically abnormal, with endothelial cells (ECs) often displaying a defective monolayer with abnormal sprouts and large intracellular holes.^26^ Tumour EC activation is primarily regulated by the angiopoietin (Ang)‐Tie2 pathway. When Tie2 is activated, Ang2 can promote angiogenesis or vascular degeneration depending on the presence or absence of vascular endothelial growth factor (VEGF).^27^ As such, VEGF is a key angiogenic factor that is upregulated in TME and induces tumour angiogenesis and abnormal neovasculature formation.^28^ This results in leaky vessels that disrupt blood flow and lead to the dysfunctional trafficking of T cells into the tumour sites.^29^ In addition, VEGF‐A, a proangiogenic factor, which regulates angiogenesis in cancer by activating vascular endothelial growth factor receptor 1 (VEGFR‐1) and VEGFR‐2, can induce T cell exhaustion, reflected by the expression of programmed cell death protein 1 (PD‐1), cytotoxic T‐lymphocyte associated protein 4 (CTLA‐4) and T‐cell immunoglobulin mucin‐3 (Tim‐3).^30^ Another unique aspect of the aberrant tumour vasculature is the expression of the Fas ligand (FasL), which is absent in normal vasculature.^31^ When the FasL present in the TME is bound to the Fas on the T cells, T cell apoptosis will be initiated,^32^ thus reducing the survival and function of CD8^+^ T cells in the TME.

In addition, the aberrant vasculature of solid tumours limits nutrients and oxygen delivery to the TME, where rapidly proliferating tumour cells have higher oxygen consumption needs, resulting in hypoxia. Hypoxia‐inducible factor 1‐α (HIF‐1α), a master regulator of numerous hypoxia‐inducible genes, can upregulate VEGF, promoting angiogenesis^33^ and enhancing tumour survivability through mitogen‐activated protein kinase/extracellular signal‐regulated kinases (MAPK/ERK) pathway.^34^ HIF‐1α has also been shown to upregulate checkpoint inhibitor programmed death‐ligand (PD‐L1), which inhibits effector T cell activity in tumours.^35^

#### 1.2.2 Vasculature normalization using CAR‐T cells

Multiple strategies have been explored to target tumour vasculature for treating solid tumours. Initial solutions aim to destroy the tumour blood vessels through anti‐angiogenic therapies that starve the tumours of oxygen and nutrients, primarily by targeting pro‐angiogenesis factors such as VEGF and their downstream pathways.^36^ However, the eradication of all tumour vessels is quite challenging and may have an off‐target effect on normal vasculature. Consequently, another more feasible novel approach, namely vasculature normalization, was developed and has recently become more prevalent.^37^ Vasculature normalization is an approach where therapeutics are designed to balance pro‐angiogenic and antiangiogenic factors as well as correct the perfusion and oxygenation of the TME for better delivery of anti‐tumour therapies. CARs targeting mouse and human VEGFR2 with scFVs from DC101 or KDR1121 antibodies have been designed with the purpose to destroy tumour vasculature.^38^ In mice models, DC101‐CAR‐T has shown increased penetration into tumour vasculature and increased antitumor responses.^38^ However, in the following clinical trial, 21 of 27 patients showed progressed disease and grade 3/4 toxicity, including nausea, vomiting and hypoxia.^39^ Several preclinical studies have also shown poor performance by DC101‐CARs unless they are provided with cytokine support, like IL‐2, or when tumour‐reactive T cells are co‐transferred.^38^, ^40^, ^41^ In addition, it has also been observed that VEGF‐A, which binds to VEGFR‐2, is upregulated in the TME and is hypothesized to further impair the CAR‐T cell function by competing with CAR‐T cells to bind to VEGFR‐2.^42^ Another target on tumour ECs, namely human prostate‐specific membrane antigen (hPSMA), has also been used as a target for CAR‐T cells and shown to recognize and ablate PSMA‐positive vessels and deplete tumour cells in mice models.^43^ Tumour endothelial markers (TEM) 1 and 8 are another two emerging cancer targets present in the stroma and neovasculature. TEM‐8 targeted CAR‐T cells can successfully block tumour neovasculature,^44^ and TEM‐1 targeted CAR‐T cells, with the employment of a tri‐lobed bi‐specific T cell engager with anti‐CD3 scFv moiety, can be activated to prevent tumour establishment in vivo.^45^ In addition, CAR‐T cells have been engineered to target the C‐type lectin domain containing 14A (CLEC14A), which is a glycoprotein highly expressed on ECs in many cancers and has been shown to efficiently reduce vascular density in the TME and inhibit tumour growth.^46^

Overall, anti‐angiogenic therapeutics are less effective than expected, presumably due to the redundancy and complexity of angiogenic pathways and the activation of compensatory mechanisms for tumour survival. Thus, other targets contributing to the aberrant vasculature, such as extracellular matrix (ECM) and ECM adhesion molecules, have also been investigated for their potency in CAR‐T therapies. For instance, CAR‐T cells targeting fibronectin splice variants containing extra domain‐B (EDB) can cause tumour vascular damage and kill tumour cells in mice models.^47^, ^48^ Additionally, αvβ3 integrin targeted CAR‐T cells containing echistatin can introduce extensive bleeding in the tumour sites while having minimal effects in normal tissues,^49^ while another αvβ3‐CAR design using scFvs from monoclonal antibody hLM609 can induce complete elimination of melanoma lesions in a murine xenograft model.^50^ In addition, endothelial Wnt/β‐catenin signalling in mouse glioma models can be induced to normalize vessels and lead to diminished tumour size by directly regulating the expression of platelet‐derived growth factor B, which is an important factor in vascular maturation.^51^ Ma et al., on the other hand, knocked out p21‐activated kinase 4 which not only corrects tumour vasculature but also increases adhesion protein expression in ECs to recruit more T cells, resulting in a better effect of CAR‐T cell immunotherapy in glioblastoma.^52^ Apart from therapeutics design, there is also a lack of proper monitoring methods to evaluate vasculature normalization and its effect on immunotherapies. More recently, Mpekris et al. have established a mathematical model to simulate and predict the TME normalization strategies that may contribute to beneficial immunotherapy outcomes.^53^ Additionally, with the development of more advanced imaging systems, for example, a LED‐based photoacoustic and ultrasound imaging system to monitor heterogeneous microvasculature in tumours,^54^ CAR‐T therapeutic effects in correcting the TME can be better evaluated in the future.

### 1.3 Enhancing the penetration of CAR‐T cells across the ECM barrier

#### 1.3.1 ECM as the barrier of CAR‐T cells into the TME

The ECM is a complex network of macromolecules including collagens, glycoproteins and proteoglycans. Besides serving as a cell scaffold, the ECM provides biochemical and biophysical cues to regulate cellular functions. The ECM is part of the TME, and its structure, composition, stiffness and degradation play critical roles in cancer progression and metastasis through mechanotransduction.^55^, ^56^, ^57^, ^58^, ^59^, ^60^ Cancer‐Associated Fibroblasts (CAFs) are a diverse population of fibroblasts that reside in the TME and play key roles in ECM deposition and remodelling.^61^, ^62^ In fact, excessive production of fibrillar ECM proteins, for example, collagen, which is a phenomenon known as desmoplasia, has been associated with CAFs to induce invasive properties of tumour cells through increasing stiffness and facilitating new types of cell‐cell^59^, ^63^ and cell‐ECM communications.^64^, ^65^, ^66^, ^67^ At the same time, CAFs can produce ECM‐remodeling enzymes, including lysyl oxidase‐like 2 and matrix metalloproteinases (MMPs), to further tune the TME for tumour growth and metastasis. More importantly, the ECM serves as a physical barrier to T‐cell infiltration. Studies have shown that higher‐density ECM can lead to a higher ratio of CD4+ to CD8+ T cells with downregulated cytotoxicity and reduced proliferation.^68^, ^69^ Consistently, T cells were shown to have higher motility and more interactions with cancer cells when the ECM is loose.^70^ In addition, transcriptomics has also shown defective T cell function when co‐cultured with mammary gland carcinoma cells on collagen 4 culture.^71^ The stiffness of the tumour increases with more ECM deposition and the reversion of tumour stiffening through inhibition of collagen crosslinking was shown to promote T‐cell migration.^72^ Moreover, tenascin‐C, which is frequently upregulated in the TME, has been shown to bind to CXCL12 in CD8+ T cells and trap them in the ECM to prevent their infiltration into tumours.^73^ As the total CD8+ T cell population decreases, the exhausted CD8+ T cell subpopulation increases in the TME with elevated collagen density, which is governed by the leukocyte‐associated immunoglobulin‐like receptor 1 pathway. The overall outcome renders tumour resistance to anti‐PD‐1/PD‐L1 treatments in clinics.^74^ However, infiltrating and non‐infiltrating T cells do not exhibit a consistent difference in their expression of chemokine receptors or adhesion molecules,^75^ suggesting that the adhesion and chemokines may play a limited role in T cell infiltration. Since CAR‐T therapies rely on the trafficking of engineered T cells to tumour sites, the ECM also serves as a physical barrier for CAR‐T cells to infiltrate and have direct contact with cancer cells. Indeed, this obstacle could be a significant limitation of CAR‐T therapy efficacy in treating solid tumours. Thus, more research is needed to fully understand how CAR‐T cells respond to and migrate in the TME in the context of the ECM.

### 1.4 Enhancing the penetration of CAR‐T cells across the physical barrier

As CAR‐T cell therapies rely on engineered T cell infiltration into the tumours and their interaction with cancer cells, examining the effects of the ECM on CAR‐T therapy efficacies is crucial. Indeed, multiple designs have been adopted in CAR‐T cell therapy to overcome such obstacles. Focuses have been on matrix‐degrading agents such as collagenases and hyaluronidases, which have been shown to improve the delivery and efficacy of a variety of cancer treatments, including anti‐tumour antibodies^76^ and chemotherapies.^77^, ^78^ While the systematic administration of matrix‐degrading agents showed promising results in animals, it may have toxicity when applied in humans as ECM is a component in most tissues. Thus, tumour‐specific delivery or cell‐specific expression of these reagents is highly recommended. Since macrophages are an essential source of MMPs, Zhang et al. engineered HER2‐CAR/CD147 macrophages, which could trigger the internal signalling of CD147. As a result, there will be an increase in the expression of MMPs, a decrease in collagen deposition after antigen binding, and ultimately an increase in T cell infiltration in vivo.^79^ GD2‐CAR has also been engineered with heparinase, which degrades heparan sulfate, another important proteoglycan in the ECM and showed an improved capacity to degrade the ECM and achieved better infiltration and killing efficiency.^80^ Studies have also targeted CAFs expressing fibroblast activation protein‐α (FAP) in the majority of cancer stroma using CARs that recognize this protein (FAP‐CAR).^81^, ^82^, ^83^, ^84^ With FAP‐CAR‐T cells, tumour vasculature density is greatly reduced and the growth of desmoplastic cancer is retained. The underlying mechanism of the inhibited tumour growth is that the FAP‐CAR‐T cells disrupted the ECM, allowing for more endogenous CD8+ T cell antitumor responses from host immunity.^83^ A phase I clinical trial (NCT01722149) with FAP‐CAR and anti‐PD1 checkpoint blockade antibody has shown persistence of the CAR‐T cells and no toxicity in patients.^84^ The initial success of CAF targeting strategies provides new insights into combining ECM‐targeting therapeutics with current immunotherapies for better treatment efficacies. Since transforming growth factor beta (TGF‐β) can act on CAFs to induce collagen production, one group showed that by administration of the TGF‐β inhibitor, SB431542, CAR‐T cell migration and cytotoxicity are restored against ovarian cancer cells.^85^ More recently, efforts have been made to remodel the ECM with nanotechnology. Chen et al. have designed a CAR‐T therapy with nano‐photosensitizer indocyanine green nanoparticles (CT‐INPs) that can modulate the microenvironment when irradiated, consequently destroying the ECM with photothermal intervention and at the same time retaining CAR‐T cell activities.^86^ Similarly, Xie et al. have engineered a heavy‐chain variable domain‐based CAR‐T cell that targets the EDB splice variant of fibronectin overly expressed in tumour ECM and neovasculature and have demonstrated a slower tumour growth rate in mice models.^48^ In summary, these ECM‐targeting strategies project greater potential for immunotherapies to synergistically modify the TME while targeting cancer cells for better efficiency and efficacy.

### 1.5 Targeting the heterogeneous tumour subpopulations

#### 1.5.1 Tumour cell heterogeneity

Recent studies have shown that most solid tumours consist of heterogeneous cell subpopulations that exhibit diverse phenotypes and genotypes. Tumour heterogeneity refers to the diverse cell subpopulations within a solid tumour and associated cancerous tissues. Three main categories of tumour heterogeneity can often be found in an individual patient, namely, cell subpopulation heterogeneity within a primary tumour (intra‐tumour), heterogeneity between a primary tumour and related metastasized sites (inter‐metastatic), and heterogeneity within metastases (intra‐metastatic).^87^ In most solid tumours intra‐tumoral heterogeneity of antigen expression in cancer cells impairs the detection of cancerous cells by CAR‐T cells, which are usually designed to target a single tumour‐associated antigen (TAA). Particularly, different tumour types and cells within the same tumour can express different TAAs with varying expression levels, making it difficult to select a proper TAA target for effective CAR designs. Moreover, normal tissues can share the same targets as tumours, triggering T cells to attack and elicit on‐target off‐tumour toxicities which can be lethal.^88^ One example is a fatal case to treat ERBB2 overexpressing tumour with HER2‐targeting CAR‐T cells which triggered severe toxicities in the patients’ lungs.^89^ From clinical trials, it is also not unusual that antigen escape or antigen loss can occur upon CAR‐T immune pressure where tumours will evolve to reduce antigen expression to avoid detection by CAR‐T cells,^90^ and this process can lead to cancer relapse. While there is no consensus on whether tumours originate from heterogeneous cell populations or develop heterogeneity during tumorigenesis, it is generally believed that multiple factors may contribute together to aggravating tumour heterogeneity.

Genetic instability is one of the most highly studied sources of tumour heterogeneity. Major sources of instability include genetic mutations, epigenetic variations and plastic gene expressions. Genetic mutations often stem from a deficiency in genome replication and repair mechanisms among other genome editing processes. These mutations lead to significant variations in the cellular genome,^91^ thus producing heterogeneous phenotypic expressions in tumour cells. Moreover, genetic mutations are not uncommon, especially in cancer cells, which have been demonstrated to have dramatically higher mutation rates than normal cells.^92^ Epigenetic variations have also been suggested to play a major role in tumour heterogeneity, especially through random epigenetic changes during tumour progression and alterations of cancer stem cells (CSCs). Many studies have shown that epigenetic changes, especially DNA methylation, occur often and chaotically during early tumour formation,^93^ and continue to occur randomly throughout tumour progression.^94^ These aberrant epigenetic changes alone could produce some levels of tumour heterogeneity. In addition, CSCs often refer to a subpopulation of tumour cells that display high self‐renewal and differentiation abilities. Many studies on CSCs have suggested that certain epigenetic changes in CSCs may produce dramatic alterations to their phenotypes, producing non‐tumorigenic, yet phenotypically heterogeneous cell subpopulations that make up the majority of a tumour.^95^ Plastic gene expression has been shown to occur in cancer cells and is another origin of tumour heterogeneity.^96^, ^97^ Studies have shown that the cellular state along with certain surface antigens of cancer cells can be reversibly altered in response to the changing of the cellular environment.^95^ The TME also plays a significant role in the divergent phenotypic expression of tumour cell subpopulations. One major example of this phenomenon is the disparate distribution of blood vessels within a tumour.^98^, ^99^, ^100^ Blood vessels transport necessary nutrients to cells and remove waste products from cells. The heterogeneous distribution of blood vessels can aggravate genomic instability through multiple factors, including hypoxia, acidosis, oxidative stress and variable hormonal and growth factor delivery.^101^ Another significant environmental influence on tumour heterogeneity is the presence of other non‐tumour cell types such as CAFs, mesenchymal stem cells (MSCs) and ECs. These cell types secrete a variety of cytokines, growth factors and ECM components that can alter the TME and contribute to the heterogeneous phenotypes of tumour cells.^102^, ^103^ For example, CAFs have been shown to secrete soluble factors and exosomes that promote “stemness” in tumour cells and CSCs,^104^, ^105^, ^106^, ^107^ which in turn enhances the ability of CSCs to promote tumour heterogeneity.

#### 1.5.2 Addressing heterogeneity of tumour cells

Multi‐valent CAR strategies aim to achieve the recognition of multiple different antigens and tumour killing using a single CAR construct.^108^, ^109^ Recently, researchers have engineered a variety of bi‐valent and tri‐valent technologies that have displayed the successful killing of tumour cells with heterogeneous antigen expression. For example, Grada et al. have developed a bi‐valent Tandem CAR (TanCAR) technology, where two different scFv molecules are connected by a flexible amino acid linker to the CAR construct, allowing for higher valency and enhanced heterogeneous tumour cell killing.^110^ Similarly, Schneider et al. have developed a tri‐valent CD19/20 and CD22 targeting CAR‐T cell by co‐expressing a tandem CD19 and CD20 CAR construct linked with a P2A linker to a CD22 CAR construct in a single CAR‐T cell.^111^ More complex systems, such as SynNotch CAR‐T cells also use multivalent CAR strategies to enhance heterogeneous tumour killing in a “priming and killing type” manner.^112^, ^113^, ^114^ Specifically, Choe et al. display that SynNotch CAR circuits, primed by epidermal growth factor receptor VIII (EGFRVIII) antigen expression in GBM cells to express a TanCAR construct, can kill EGFRVIII negatively expressing tumour cells through a “trans‐killing” mechanism, where a CAR protein is primed to be expressed byEGFRVIII positive tumour cells and can attack and kill EGFRVIII negative tumour cells in the local neighbourhood expressing a different antigen.^112^

Another strategy to address tumour antigen heterogeneity is by utilizing adapter molecules to simultaneously recognize multiple tumour antigens with a single CAR construct. In general, these strategies employ an adapter molecule containing a tumour‐specific binding domain and a common adapter domain, which can bind to a CAR construct engineered with an adapter binding domain to activate the CAR‐T cell and induce tumour‐cell killing. For example, Urbanska et al. and Lohmeuller et al. have developed “universal” tumour‐targeting strategies using biotinylated antibodies adapter molecules, which can bind to various target cancer cells, combined with CAR constructs containing avidin or streptavidin domains.^115^, ^116^ It has been shown that a variety of tumour‐targeting antibodies can be engineered into the biotinylated‐antibody adapter molecule such that a single avidin or streptavidin CAR construct can bind and kill various targets if the biotinylated antibody is bound to the tumour cell.^115^, ^116^ Lee et al. used a similar approach by employing fluorescein linked to tumour‐specific ligands adapter molecules combined with CAR constructs expressing anti‐fluorescein binding domains.^117^ Kudo et al. have shown that simply engineering a CAR‐T cell expressing a CD16 receptor CAR and anti‐TAA antibodies can also enable CAR‐T cell killing of multiple different antigen‐expressing tumour cells.^118^ In addition, another interesting engineering approach utilized oncolytic viruses to deliver truncated CD19 (OV19t) de novo at the tumour cell surface to achieve more CD19‐CAR T targeting and subsequent killing. Furthermore, the usage of an oncolytic virus also propagates the killing by attracting more endogenous or adoptively transferred T cells in vivo.^119^ Another recently developed system by Cho et al. named split, universal and programmable (SUPRA)‐CAR uses leucine zipper dimerizing motifs to couple the CAR constructs (zipCAR) and the tumour‐targeting scFVs (zipFV) to target multiple antigens.^120^ This system is further enhanced by the incorporation of antigen‐binding logic gates to reduce tonic signalling and non‐specific T‐cell activation. Choi et al. have developed a novel approach to target heterogeneous antigen expression in Glioblastoma tumours by using a CAR construct specific for EFGRVIII and a bispecific T‐cell engager (BiTE) specific for EGFR.^121^ This technology uses the CAR construct to locally deliver BiTE molecules, which are bispecific antibodies for EGFR and T‐cells, that activate bystander T cells to kill EGFR‐expressing tumour cells. Their studies have displayed almost complete killing of heterogeneous tumours in glioblastoma mouse models.

## 2 OVERCOMING THE IMMUNOSUPPRESSIVE MICROENVIRONMENT

### 2.1 Immunosuppressive cells and cytokines inhibit the function of CAR‐T cells

The immunosuppressive TME presents several challenges to CAR‐T cell therapy. The CAR‐T cell activity in the immunosuppressive TME can be inhibited by immune inhibitory ligands on the tumour cell surface,^122^ which could induce T cell exhaustion, immunosuppressive cells, and immunosuppressive cytokines.^123^ Major immunosuppressive cells include regulatory T cells (Treg), tumour‐associated macrophages (TAM) and myeloid‐derived suppressor cells (MDSC). Tregs secrete immunosuppressive cytokines which inhibit cytotoxic T cell function or limit the number of IL‐2 that can activate cytotoxic T cells.^124^ Similar to Tregs, MDSCs^125^, ^126^ and TAMs^127^, ^128^ were found to negatively modulate the function of T cells in the TME by modulating tumour vasculature or pre‐metastatic niche formation, producing various anti‐inflammatory cytokines, and inducing epithelial‐mesenchymal transition (EMT) of tumour cells.^125^, ^126^, ^127^, ^128^ For instance, Cervantes et al. demonstrated that the co‐culture of CAR‐T cells with MDSCs could reduce the T cell production of IFNγ and inhibit the CAR‐T cell activation.^129^ Cytokines secreted in the TME can also contribute to its immunosuppressive effect. For example, tumour ECs can produce angiocrine factors to promote angiogenesis, such as IL‐6, which converts TAMs from a pro‐inflammatory M1 phenotype to an immunosuppressive M2 phenotype. The M2 phenotype has been shown to produce more anti‐inflammatory cytokines, such as tumour growth factor‐β (TGF‐β) and IL‐10,^130^ which inhibits T cell survival and activation.^131^ Additionally, crosstalk between tumour ECs and MDSCs through VEGF can also negatively impact anti‐tumour responses and prompt angiogenesis.^132^ Studies have also recently shown that sustained antigenic stimulation, inhibitory receptors, immunosuppressive factors and cells, and transcription factors are major contributors to T cell exhaustion.^133^ Another important contributor to the overall immunosuppressive TME is hypoxia, which has multiple functions, including inducing angiogenesis, triggering EMT, maintaining cancer stem cells and regulating immune cell populations in tumour immunity. Research has found that low oxygen significantly depresses effector T lymphocytes differentiation, proliferation, and killing functions and leads to decreased IFN‐γ and IL‐2 secretion.^134^, ^135^ Furthermore, constitutive expression of HIF‐1 and HIF‐2 can delay CD8+ T cell differentiation into effector cells, but also increase their cytotoxic functions.^134^ Additionally, hypoxic stress promotes local immune suppression by driving MDSCs with higher PD‐L1 expression to accumulate in the TME,^136^ inducing Treg cell formation through the binding of HIF‐1 to the forkhead box P3 (Foxp3) promoter region in CD4+ T cells,^137^ and attracting Tregs to the TME through the increased secretion of chemokine (C‐C motif) ligand 28 (CCL28) by ovarian tumour cells.^138^ In addition, hypoxia can cause an elevated level of adenosine in the TME which induces A2A adenosine receptor (A2AR)‐ and A2B adenosine receptor (A2BR)‐mediated intracellular cAMP build‐up in T cells and impairs T cell function.^139^ Specifically for CAR‐T cells, hypoxia has been shown to reduce CAR‐T cell expansion and differentiation, thus increasing the CD4:CD8 ratio and reducing granzyme B production.^140^

### 2.2 Overcoming the immunosuppressive microenvironment

Two commonly used strategies for efficient immunotherapy include targeting immunosuppressive cell types and cytokines. Recently, Brog et.al showed that CAR‐T cells armoured with the interleukine‐2 superkine (Super2) and interleukine‐33 can promote antitumor efficiency in multiple solid tumour models.^141^ Other studies have shown that IL‐18‐secreting CAR‐T cells have enhanced functionality by altering the inflammatory TME and the immune cell landscape inside solid tumours.^142^, ^143^ These IL‐18 armoured CAR‐T cells were engineered and found to exhibit superior activity against large pancreatic and lung tumours. Such effects were found to be associated with the depletion of the inhibitory M2‐polarized macrophages and Treg cells and the recruitment of proinflammatory M1‐polarized macrophages,^144^ via IL‐18 autocrine stimulation.^145^ In addition to IL‐18, CAR‐T cells that were engineered to secrete IL‐12 were also shown to have enhanced antitumor activity with increased resistance to Treg‐mediated inhibition, increased cytotoxicity,^146^ and significantly improved cell expansion.^147^, ^148^ However, while the use of pro‐inflammatory cytokines could be effective in combating Tregs, their harmful side effects must be considered when implementing these strategies.^149^ For instance, IL‐18 was reported to promote tumour progression by inducing angiogenesis, immune escape and metastasis.^150^ In addition, the overexpression of IL‐18 may disrupt the balance of IL‐18 and its inhibitor protein, IL‐18 Binding Protein (IL‐18BP), which may lead to severe diseases such as sepsis and acute kidney injury.^151^ Furthermore, the systematic increase of IL‐12 may cause a rapid increase of other pro‐inflammatory cytokines, such as IFN‐γ, tumour necrosis factor‐α (TNF‐α) and IL‐6,^152^ which can be life‐threatening.^153^ Work must be done to establish confirmed safe amounts of IL‐18 and IL‐12 that will still be effective but not harmful.

Engineering CAR‐T cells to be less sensitive to the immunosuppressive cytokines is another strategy to mitigate immunosuppressive cytokines‐mediated immunosuppression. For example, TGFβ is one of the most well‐known factors that exert systemic immune suppression by directly suppressing T cell cytolysis,^154^, ^155^, ^156^ and inducing the differentiation of T cells into the inhibitory regulatory phenotype.^156^, ^157^ To overcome the inhibitory effects of TGFβ on CAR‐T cells, a TGFβ dominant‐negative receptor (TGFβ DNR), which renders transduced cells unresponsive to TGFβ, was engineered to be overexpressed in CAR‐T cells targeting PSMA.^158^ The TGFβ DNR PSMA‐CAR‐T cells have shown increased proliferation, enhanced cytokine secretion, resistance to exhaustion, long‐term in vivo persistence and efficient induction of tumour eradication in the preclinical study.^158^ In addition, clinical application of these TGFβ‐resistant CAR‐T cells has been shown feasible and generally safe when targeting metastatic castration‐resistant prostate cancer (mCRPC).^159^ However, in a recent report, TGFβ DNR PSMA‐CAR‐T cells were found to cause fatal toxicities including immune effector cell‐associated neurotoxicity syndrome and multi‐organ failure in two mCRPC patients out of nine, and the exact mechanisms of these immune‐mediated toxicities are still unclear.^160^ The emerging CRISPR technology makes the precise genetic manipulation of CAR‐T cells possible. It has been shown that knocking out the endogenous TGF‐β receptor II in CAR‐T cells using CRISPR/Cas9 could reduce the Treg conversion and prevent the exhaustion of CAR‐T cells, hence, having better in vivo tumour elimination efficacy.^161^ To convert TGF‐β from an immunosuppressive cytokine to a strong stimulant in T cells, TGF‐β CAR‐T cells were developed, which not only inhibits endogenous TGF‐β signalling in T cells but also converts TGF‐β into a potent T‐cell stimulant.^162^ These TGF‐β CAR‐T cells were later shown could significantly improve the anti‐tumour efficacy of neighbouring cytotoxic T cells.^163^ A similar strategy was employed to reduce CAR‐T cell sensitivity to immunosuppressive adenosine by blocking A2AR through CRISPR gene knockout or pharmacological targeting. After A2AR blockage CAR‐T cells showed higher anti‐tumour efficacy.^164^ While there is still a need for CAR‐T cell therapies directly against hypoxic TME, an interesting approach utilizing the hypoxic TME was designed such that CAR‐T cells could sense hypoxia and only express CAR molecules under the hypoxic TME. For example, Juillerat et al. generated oxygen‐sensitive CAR‐T cells by fusing the HIF‐1α subdomain to CAR scaffold,^165^ and Kosti et al. designed a hypoxia‐sensing CAR by appending the oxygen‐dependent degradation domain of HIF‐1α onto the CAR while adding nine consecutive hypoxia‐responsive elements in the CAR promoter.^166^

### 2.3 Mitigate T‐cell exhaustion in the immunosuppressive TME

T cell exhaustion is another hallmark of the TME's barrier to CAR‐T therapy. T cell exhaustion is often reflected by the upregulation/co‐expression of inhibitory receptors, major changes in T cell receptor and cytokine signalling pathways, altered expression of genes related to chemotaxis and adhesion, altered expression of transcription factors and metabolic deficiencies. All of these eventually lead to weakened T cell persistence, anti‐tumour activity and overall decreased effectiveness of CAR‐T cell therapies.^133^ Several approaches to mitigate T cell exhaustion have been developed and will be summarized in the following section.

### 2.4 Targeting the inhibitory receptors

**FIGURE 3:**
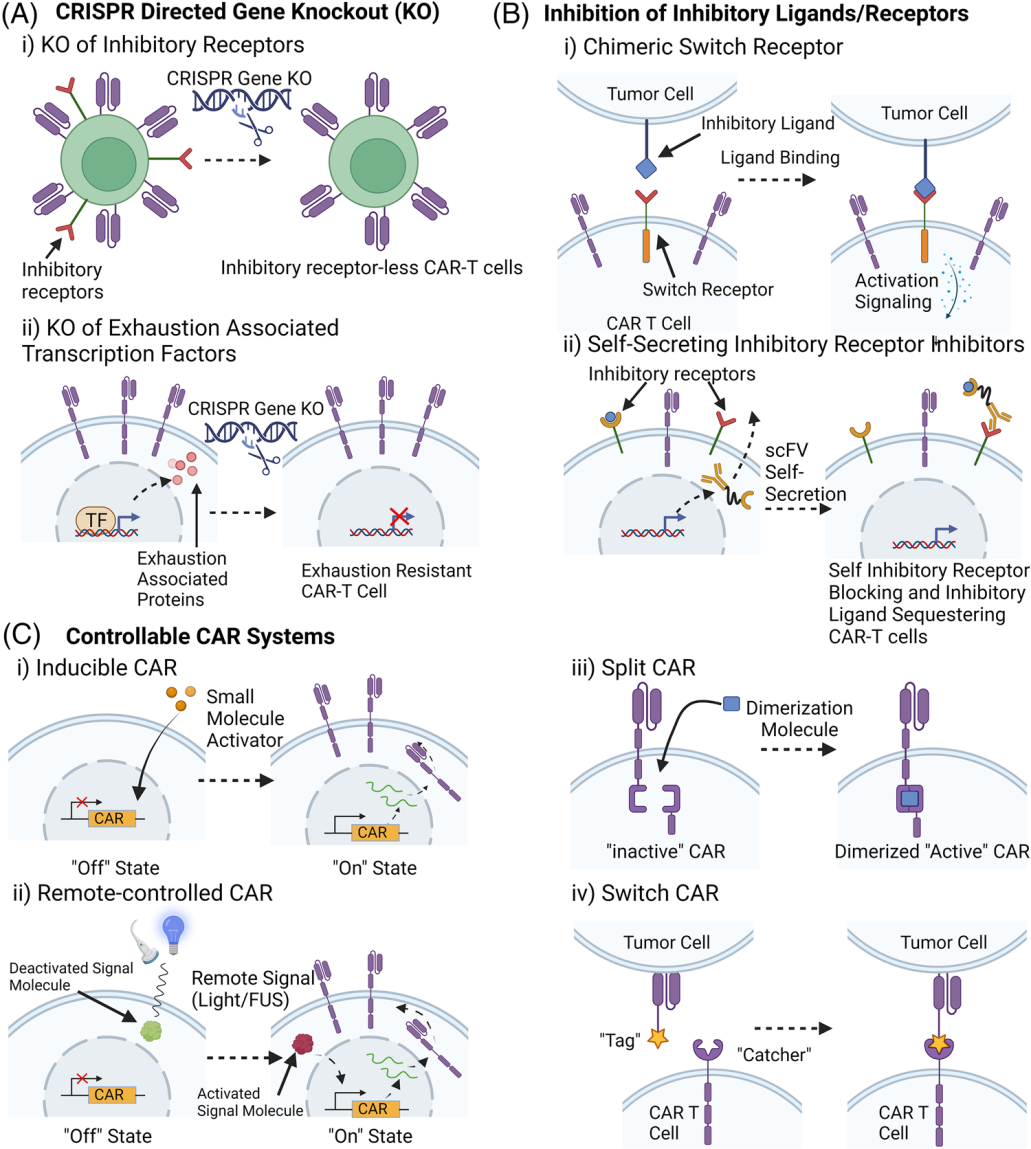
Examples of different strategies to mitigate chimeric antigen receptor (CAR) T cell exhaustion. (A) CRISPR‐cas9 gene knockout strategies to engineer exhaustion‐resistant CAR T cells. (i) Knockout of inhibitory receptors such as PD‐1, CTLA‐4, TIM‐3, LAG‐3, TIGIT and TGF‐β receptors. (ii) Knockout of exhaustion‐associated transcription factors such as SOX4, ID3 and NR4A family factors. (B) Inhibition of inhibitory receptors on CAR T cells. (i) Chimeric Switch receptors such as PD1CD28 or PDmut7R convert inhibitory ligand binding into activation signalling. (ii) CAR T‐cell self‐secretion of anti‐inhibitory receptor scFVs and/or inhibitory ligand sequestering molecules. (C) Examples of Controllable CAR Systems. (i) Inducible CAR system where CAR expression is induced by the presence of small molecular activators such as Doxycycline. (ii) Light or Focused Ultrasound (FUS) activated CAR expression (iii) Split CAR system where the CAR molecule is split into two distinct portions such that the CAR will only be functional in the presence of a dimerization molecule. (iv) Switch CAR system where the scFV contains a “tag” domain while the signalling domain contains a “catcher”. The “Tag” and “Catcher” can bind to each other to produce a functional CAR molecule

Many strategies to combat T cell exhaustion through CAR‐T cell engineering have focused on ameliorating the effects of inhibitory receptors found on T cells. One highly studied method and successful strategy is the alternation of PD‐1 interactions. PD‐1 plays a vital role in the regulation of T‐cell exhaustion. Importantly, many tumour cells present an inhibitory protein programmed death‐ligand 1(PD‐L1), which can bind to PD‐1 on CAR‐T cells and induce exhaustion in anti‐tumour T cells.^167^ One strategy used by Rupp et al. is to employ CRISPR‐Cas9 technology to delete PD‐1 in anti‐CD19 CAR‐T cells^167^ (Figure 3A). In this study, PD‐1 deleted CAR‐T cells display enhanced anti‐tumour efficacy and reduced exhaustion against both PD‐L1 positive and negative CD19+ tumour cells in vitro and in vivo.^167^ Another novel system combating the PD‐1 inhibitory pathway was developed by Liu et al. where a “PD1CD28” chimeric switch receptor engineered from the fusion of the extracellular domain of PD1 and the transmembrane and cytoplasmic signalling domain of CD28 was expressed in CAR‐T cells to convert inhibitory PD‐1/PD‐L1 signalling into CD28 T‐cell activation signalling. The expression of the PD1CD28 receptor in CAR‐T cells led to significant tumour volume reduction in solid tumours owing to reduced exhaustion, attenuation of inhibitory receptors and increased tumour infiltration^168^ (Figure 3B). This system was further improved by Huang et al. to employ a similar switch receptor (PDmut7R) where the costimulatory domain of IL‐7 is used rather than CD28 to enhance B7‐H3‐specific CAR‐T cells.^169^ The chimeric PD‐1 engineered CAR‐T cells displayed an increased secretion of cytokines, expanded populations of effector T cells, stronger anti‐tumour activity and enhanced protection against re‐challenged tumours. Rather than altering PD‐1 on CAR‐T cells, Rafiq et al. have also successfully coexpressed a PD‐1 blocking scFV in CAR‐T cells that is secreted into the local environment to block PD‐1 on CAR‐T cells and bystander tumour‐specific T cells.^170^ This study displayed enhanced anti‐tumour activity in vitro and in vivo, outperforming currently used CAR‐T cells and PD‐1 blocking monoclonal antibody treatments, thus highlighting the importance of localized delivery of PD‐1 blockers.^170^ A similar approach was developed where engineered CAR‐T cells coexpress a bispecific combined anti‐PD‐1 scFV and TGF‐β binding protein to inhibit PD‐1 receptors as well as sequester TGF‐β from the local TME^171^ (Figure 3B). The combination of PD‐1 blockade and trapping of TGF‐β allows for increased proliferation and improved effector cytokine secretion in CAR‐T cells.^171^ Moreover, this system ameliorates exhaustion in CAR‐T cells by significantly limiting PD‐1 expression and other exhaustion‐associated immune‐checkpoint molecules such as lymphocyte activation gene‐3 (LAG‐3) and TIM‐3.^171^ These PD‐1 targeting approaches were proven to significantly enhance the potency of the CAR‐T based therapy. Although not highly studied yet, similar strategies could be potentially employed against other T cell inhibitory receptors such as CTLA‐4, TIM‐3, LAG‐3 and T cell immunoreceptor with Ig and ITIM domains (TIGIT) receptors in T cells.^172^, ^173^

#### 2.4.1 Identify factors in T cell exhaustion and promote T cell performance by genetic modification

As classical inhibitory receptor targeting draws more attention, more extensive efforts have been made to unravel the intracellular mechanism underlining T cell exhaustion, especially in identifying factors that play critical roles in driving and regulating the T cell exhaustion phenotype. Combined with powerful bioinformatics and/or CRISPR‐based screening tools, several epigenetic factors were identified to be beneficial for exhaustion when over‐expressed or knocked down, which has gradually become a more prevalent direction of mitigating T cell exhaustion. Fraietta et al. reported that in a special clinical case, CAR vector integration disrupted the methylcytosine dioxygenase TET2 gene, which leads to clonal expansion of CD19 CAR‐T cells and enhanced tumour remission.^174^ This study demonstrated that the modification of key genes in T cells could enhance persistence and anti‐exhaustion. Recently, studies have shown that the overexpression of either c‐Jun, an activator protein‐1 (AP‐1) family transcription factor,^175^ or basic leucine zipper ATF‐like transcription factor (BATF)^176^ could prevent and rescue CAR‐T cells from exhaustion. However, a recent study from Zhang et.al showed knockout (KO) of BATF CAR‐T cells could enhance antitumor activity and found that BATF KO in CAR‐T cells make the cells more resistant to exhaustion and induces the formation of more central memory CAR‐T cells.^177^ These contradictory results may result from the use of different T cell donors, tumour models, or experiment conditions. Other studies have focused on the CRISPR/Cas9‐based knockout of transcription factors in CAR‐T cells such as SRY‐box transcription factor 4 (SOX4) and inhibitor of DNA binding 3 (ID3),^178^ de novo DNA methyltransferase 3 alpha (DNMT3A),^179^ or the triple knockout of nuclear receptor subfamily 4A (NR4A) family nuclear transcription factors NR4A1 (also known as NUR77), NR4A2 (NURR1) and NR4A3 (NOR1),^180^ which all have shown enhanced CAR‐T resistance to exhaustion (Figure 3A).

The flourishing of CRISPR/Cas9‐based technology provides scientists with a more powerful tool that enables active searching for related factors in vitro and in vivo for T cell exhaustion modulation. Although some of the screenings are conducted on T cells instead of directedly on CAR‐T cells, these screenings revealed candidates that can be potentially manipulated to have benefits for CAR‐T therapy. For instance, Wei et al. applied CRISPR knock‐out library screening by targeting metabolic regulators in an in vivo model and found that Regnase‐1 KO CD19 CAR‐T cells have better therapeutic performance.^181^ Related research also reported that disrupting the interaction between Roquin‐1 and Regnase‐1 strengthened anti‐tumour responses.^182^ Shifrut et al. applied genome‐wide CRISPR screening in primary human T cells and identified multiple negative regulators, including suppressor of cytokine signalling 1, transcription elongation factor B polypeptide 2, RAS p21 protein activator 2 (RASA2) and cbl proto‐oncogene B,^183^ and ablation of these targets enhanced both proliferation and in vitro anti‐cancer function of T cells. In a later work, CRISPR activation (CRISPRa) and interference (CRISPRi) screens focusing on genes related to T cell activation were performed, further illustrating that gain and loss‐of‐function screening can be combined for a more comprehensive regulator identification.^184^ Similarly, Dong et al. conducted CRISPR screening in CD8 T cells based on mouse triple‐negative breast cancer (TNBC) models and revealed that DEAH‐box helicase 37 (Dhx37) modulates T cell function, which, when knocked out, enhances the T cell activation and effector function by modulating NF‐κB signalling.^185^ Another work from the same group screened for boosters of T cell function using dead‐guide RNA (dgRNA)‐based CRISPR activation screening technology and illustrated overexpression of proline dehydrogenase 2 (PRODH2/Prodh2) in CAR‐T cells can improve antitumor efficacy in vivo.^186^ Belk et al combined in vivo and in vitro CRISPR screening, in which chromatin remodelling complex canonical barrier‐to‐autointegration factor (cBAF) and INO80 complex ATPase subunits are found to be related to T cell exhaustion and disrupting one of the cBAF complex subunits gene AT‐rich interactive domain‐containing protein 1A enhances persistence of human T cells.^187^ Through multiple genome‐wide CRISPR knock‐out screens under different immunosuppressive conditions, Carnevale et al. found the ablation of RASA2, a RAS GTPase‐activating protein, could enhance the MAPK signalling and CAR‐T cell cytolytic activity.^188^ Aside from CRISPR‐based screening strategies, Legut et al. utilized a genome‐scale gain of function screening by introducing human open reading frames library into T cells and found that lymphotoxin‐β receptor, a hit typically expressed in myeloid cells but not T cells, can enhance effector function and exhaustion resistance.^189^ Overall, the recent developments in high throughput genomic screening technologies employing CRISPR gene KO and CRISPR activation have allowed for the efficient in vivo screening of potential therapeutic targets to ameliorate CAR‐T cell exhaustion.

#### 2.4.2 Design remote controllable CAR‐T cells

Engineering approaches for the CAR construct have also been employed to mitigate CAR‐T cell exhaustion. Many controllable CAR systems have been employed to diminish the over‐activation of CAR‐T cells and provide controlled resting periods for CAR‐T cells (Figure 3C). Some researchers have developed drug‐stabilized and drug‐destabilized CAR molecules, which allow for controllable CAR activation and deactivation based on the presence or absence of certain small‐molecule drugs.^190^, ^191^ Similar controllable strategies such as Tet‐on/Doxycycline^192^ controlled CAR expression and light controllable systems^193^ can also be explored as potential venues to reduce CAR‐T cell exhaustion (Figure 3C). Other controllable strategies that decouple CAR antigen recognition from T cell activation can also be used to mitigate T cell exhaustion. Akin to small molecule‐controlled systems, Liu et al. have developed a switchable CAR system that employs a molecular “switch” that can recognize a TAA on the tumour cells and binds to the CAR construct to activate the CAR‐T cell^194^ (Figure 3C). Split CAR technology can be used in a similar manner, where the dimerization of the CAR construct is controlled by the presence of a dimerization molecule^194^ (Figure 3C). A similar “switch” idea was achieved by a protease‐based CAR design, in which hepatitis C virus NS3 protease was used to cut CAR and terminate CAR signalling, achieving “on and off” control using a protease inhibitor. Indeed, the protease‐controlling CAR‐T cells are less exhausted^195^ after CAR‐T manufacturing without the protease inhibitor and more functional when induced by protease inhibitor for 24 hours, compared to constitutive CAR. Another engineering strategy is the application of high‐performance costimulatory domains in CAR constructs. For example, Long et al. demonstrated that the application of a 4‐1BB costimulatory domain rather than a CD28 domain in CD19 CAR was able to greatly reduce the expression of T cell exhaustion markers.^196^ In addition, remote non‐invasive controllable systems have been developed utilizing light^193^, ^197^ or ultrasound^198^ to control the expression of CAR molecules on the cell surface (Figure 3C). These approaches should allow remote‐controlled, noninvasive gene activation with high spatial and temporal precision for the delivery of immunotherapy to tumours. Among these methods, focused ultrasound controllable CAR‐T cells are of great potential to be further applied clinically, as the ultrasound can penetrate any depth inside of the human body, utilizing the clinically available focused ultrasound instrument. In a recent study, Wu et al. showed that ultrasound could be used to remotely focus the energy and rapidly generate heat in the target cells and tissues at local sites with high precision to control the CAR molecule expression inside tumours to achieve efficient tumour clearance.^198^

## 3 CONCLUSION

**FIGURE 4:**
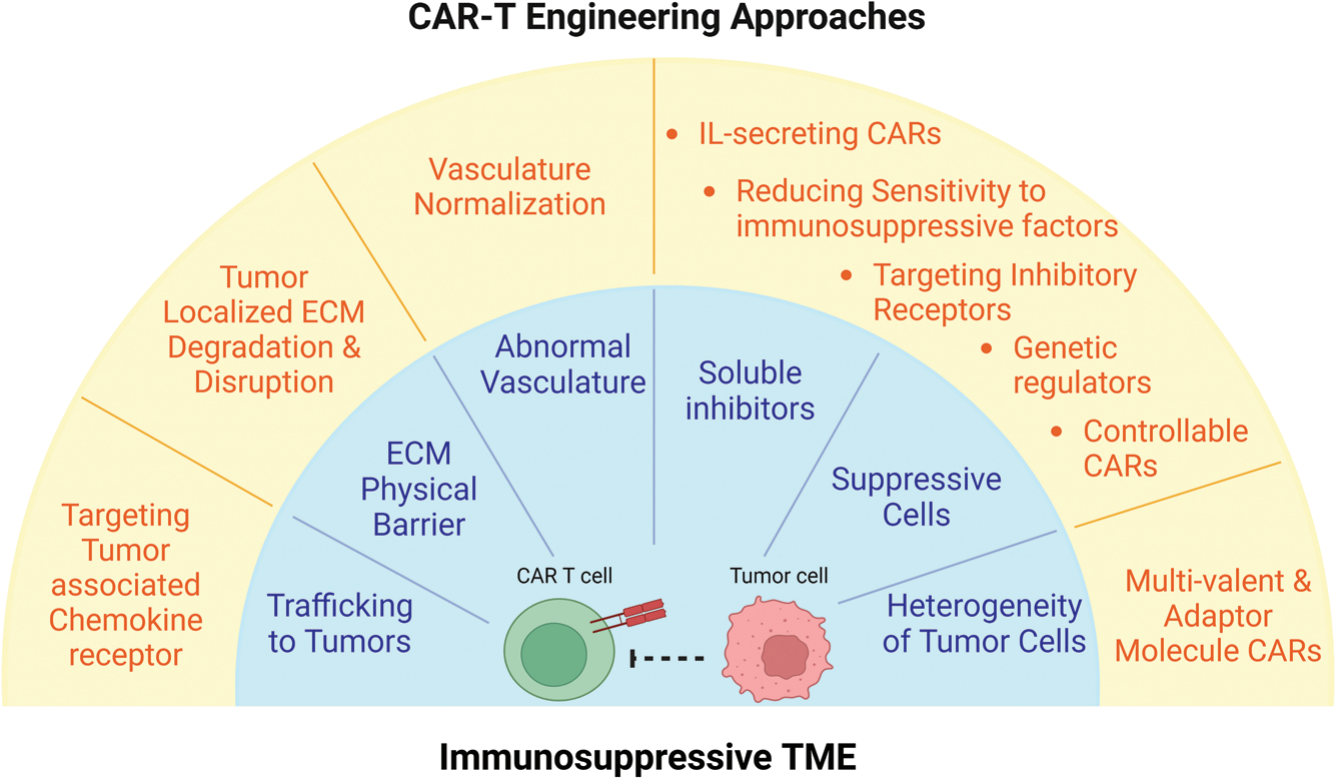
Summary of major bottlenecks in solid tumour chimeric antigen receptor (CAR) T cell therapy and solutions to overcome them. A detailed summary table can be found in the Table S1

In this review, we have summarized the current bottlenecks and the engineering approaches to improve the efficiency of the CAR‐T cell for solid tumours (Figure 4) which has also been summarized in Table S1. We have to admit that CAR‐T cell engineering is an emerging and fast‐growing field, thus we may not cover all the aspects in this review paper. Furthermore, while these engineering approaches we summarized here are of great potential to be applied clinically, challenges remain before CAR‐T cells can be generally applicable to patients with solid tumours. We can foresee that utilizing the remote and non‐invasive controllable system such as ultrasound to control the activity of CAR‐T cells to reduce the toxicity and exhaustion of CAR‐T cells is of great potential for CAR‐T cells to be applied in solid tumour treatment. In addition, elucidating the bidirectional interactions of CAR‐T cells with tumour cells in its microenvironment is critical for designing next‐generation CAR‐T cell therapy for solid tumours. Single‐cell‐based sequencing provides unprecedented opportunities to reveal the dynamics of transcriptome and epigenome; however, these data are a snapshot of the cell state at a given time.^199^ Furthermore, the regulators identified from different studies do not act in isolation but instead orchestrate a cross‐regulated and highly coordinated signalling network to control the interactions between cancer and immune cells; however, this interaction is not clear. The integration of live cell imaging and single‐cell omics analysis of CAR‐T cells should provide this missing link in these studies. We can foresee that live‐cell imaging would provide more detailed information on the cell signalling dynamics with a high spatiotemporal resolution, and fluorescent protein (FP)‐based biosensors of signalling molecule activities could be a powerful tool to study these key regulators and enable direct interrogation of their activity in native biological contexts with ultra‐sensitivity and high spatiotemporal resolution.^60^, ^200^ Thus, parallel examination of the key node regulators simultaneously in cancer‐immune interacting environments should be needed to reveal novel insights into the systematic behaviours and identify essential links for therapeutic manipulation. With the rapid development of CRISPR and its related technologies in genome editing and reprogramming, it is also expected that genome‐wide screenings will reveal more potent regulators of CAR‐T functions, which can be reprogrammed to enhance therapeutic efficacy and safety.

In summary, CAR‐T cell therapy is becoming a paradigm‐shifting therapeutic approach for cancer treatment and is one of the most promising therapeutics for the treatment of solid tumours. The leverage of technological advancements in different areas and their application in the next‐generation CAR‐T cell design to overcome the solid tumour microenvironment should greatly benefit the treatment of cancer patients.

## CONFLICT OF INTEREST

Yingxiao Wang is a scientific co‐founder of Cell E&G Inc and Acoustic Cell Therapy Inc. These financial interests do not affect the design, conduct, or reporting of this article.

## Supporting information



Supporting Information
