# Supplementary material for: Engineering chimeric antigen receptor T cells for solid tumour therapy
Source: Clin Transl Med. 2022 Dec 10;12(12):e1141. doi: 10.1002/ctm2.1141 (PMC9736813; doi:10.1002/ctm2.1141)
Supplement: Supplementary file 1 — Supporting Information [file CTM2-12-e1141-s001.docx]

**Supplementary file**

**Engineering CAR-T Cells for Solid Tumor Therapy**

Longwei Liu^1#^*, Yunjia Qu^1#^, Leonardo Cheng^1#^, Chi Woo Yoon^1^, Peixiang He^1^, Abdula Monther^1^, Tianze Guo^1^, Sarah Chittle^1^, Yingxiao Wang^1^*

1. Department of Bioengineering, Institute of Engineering in Medicine, University of California, San Diego, 9500 Gilman Drive, La Jolla, CA, 92093-0435, USA

# These authors contributed equally.

* Corresponding authors:

Yingxiao Wang, [yiw015@eng.ucsd.edu](mailto:yiw015@eng.ucsd.edu);

Longwei Liu, lol001@eng.ucsd.edu

**Table 1: Summary of Major Bottlenecks in Solid Tumor CAR-T cell Therapy and Solutions to overcome them.**

| **Major**  **Bottlenecks** | **Example Solutions** | **CAR-T design** | **Relevant References** |
| --- | --- | --- | --- |
| T cell homing and penetration | Targeting Tumor associated Chemokines using Chemokine receptor engineering CAR T cells | CCR4-expressing CD30 CAR | [Di Stasi et al., 2009](#_ENREF_45) |
|  |  | CCRb2-expressing GD2 CAR | [Craddock et al., 2010](#_ENREF_38) |
|  |  | CCRb2-expressing mesoCAR | [Moon et al., 2011](#_ENREF_122) |
|  |  | CXCR1/CXCR2-expressing αvβ6-targted CAR | [Whilding et al., 2019](#_ENREF_186) |
|  |  | CXCR1/CXCR2-expressing CD70 CAR | [Jin et al., 2019](#_ENREF_79) |
|  |  | CXCR2-expressing GPC3 CAR | [Liu et al., 2020a](#_ENREF_104) |
|  | Vasculature Normalization | Anti-DC101 CAR | [Chinnasamy et al., 2010](#_ENREF_31) |
|  |  | Anti-KDR1121 CAR | [Chinnasamy et al., 2010](#_ENREF_31) |
|  |  | Anti-hPSMA CAR | [Santoro et al., 2015](#_ENREF_158) |
|  |  | Anti-TEM-8 CAR | [Byrd et al., 2018](#_ENREF_16) |
|  |  | Anti-TEM-1 CAR | [Fierle et al., 2021](#_ENREF_55) |
|  |  | Anti-CLEC14A CAR | [Zhuang et al., 2020](#_ENREF_197) |
|  |  | Anti-EDB CAR | [Wagner et al., 2021](#_ENREF_179); [Xie et al., 2019](#_ENREF_190) |
|  |  | Anti-αvβ3 integrin CAR | [Fu et al., 2013](#_ENREF_57); [Wallstabe et al., 2018](#_ENREF_180) |
|  | Tumor Localized ECM Degradation/Disruption | HPSE-expressing GD2 CAR | [Caruana et al., 2015](#_ENREF_21) |
|  |  | Anti-FAP CAR | [Curioni et al., 2019](#_ENREF_40); [Kakarla et al., 2013](#_ENREF_83); [Schuberth et al., 2013](#_ENREF_162); [Wang et al., 2014](#_ENREF_181) |
|  |  | Anti-EDB CAR | [Wagner et al., 2021](#_ENREF_179); [Xie et al., 2019](#_ENREF_190) |
| Heterogeneous Tumor Subpopulations | Multi-valent CARs | CD19-HER2 Tan CAR | [Grada et al., 2013](#_ENREF_62) |
|  |  | BCMA/CS1 Tan CAR | [Zah et al., 2020](#_ENREF_194) |
|  |  | Tri-valent CD19/20 and CD22 CAR | [Schneider et al., 2021](#_ENREF_161) |
|  |  | EGFRvIII (synNotch priming) EphA2/ IL13Rα2 Tan CAR | [Choe et al., 2021](#_ENREF_35) |
|  | Adapter Molecule CARs | Biotinylated antibodies/ Monomeric streptavidin 2 (mSA2) biotin-binding domain-CAR | [Lohmueller et al., 2018](#_ENREF_112) |
|  |  | Biotinylated antibodies/ extracellular-modified avidin CAR | [Urbanska et al., 2012](#_ENREF_175) |
|  |  | Adapter molecule with FITC /anti-FITC CAR | [Lee et al., 2019](#_ENREF_95) |
|  |  | Humanized antibodies (e.g., trastuzumab)/High-affinity CD16 (FCGR3A) V158 variant CAR | [Kudo et al., 2014](#_ENREF_91) |
|  |  | SUPRA CAR (zipCAR and zipFv) CAR | [Cho et al., 2018](#_ENREF_34) |
| Immunosuppressive Microenvironment | Interleukin secreting CARs | Interleukin-18 (IL-18)- Armored CAR | [Hu et al., 2017](#_ENREF_71); [Rafiq et al., 2020](#_ENREF_147) ;[Chmielewski and Abken, 2017](#_ENREF_33) |
|  |  | Interleukin-12 (IL-12) Armored CAR | [Pegram et al., 2012](#_ENREF_141) |
|  |  | Superkine IL-2 and IL-33 Armored CAR | [Brog et al., 2022](#_ENREF_13) |
|  | Mitigate inhibitory effects of hypoxia | A2AR-KO HER2 CAR | [Beavis et al., 2017](#_ENREF_7) |
|  |  | HIF1α-inducible CAR | [Juillerat et al., 2017](#_ENREF_81); [Kosti et al., 2021](#_ENREF_88) |
|  | Reducing sensitivity to immunosuppressive factors | TGFβ dominant-negative receptor (TGFβ DNR) Armored CAR | [Kloss et al., 2018](#_ENREF_88); [Narayan et al., 2022](#_ENREF_131) |
|  |  | TGF-β receptor II KO CAR | [Tang et al., 2020](#_ENREF_174) |
|  |  | Anti-TGF-β CAR | [Chang et al., 2018](#_ENREF_24); [Hou et al., 2018](#_ENREF_71) |
|  | Targeting Inhibitory Receptors | PD-1 KO CAR T | [Rupp et al., 2017](#_ENREF_155) |
|  |  | Anti-B7-H3 CAR | [Huang et al., 2020a](#_ENREF_74) |
|  |  | PD-1-blocking scFv | [Rafiq et al., 2018](#_ENREF_149) |
|  |  | bispecific trap protein co-targeting PD-1 and TGF-β | [Chen et al., 2021a](#_ENREF_27) |
|  |  | PD-1 Chimeric Switch Receptors | Liu et al., 2016; Huang et al., 2019 |
|  | Modification of genetic regulators of CAR-T Cells | Gene KO of SOX4; ID3; DNMT3A; NR4A; Regnase-1; Dhx37; Arid1A; BATF | [Good et al., 2021](#_ENREF_62); [Prinzing et al., 2021](#_ENREF_147); [Chen et al., 2019](#_ENREF_25); [Wei et al., 2019](#_ENREF_186); [Dong et al., 2019](#_ENREF_50); [Belk et al., 2022](#_ENREF_10);Zhang et al.,2022 |
|  |  | Over expression of c-Jun; BATF; PRODH2; LTBR | [Lynn et al., 2019](#_ENREF_116);[Seo et al., 2021](#_ENREF_165); [Ye et al., 2022](#_ENREF_193); [Legut et al., 2022](#_ENREF_97) |
|  | Controllable CARs | Small molecule drugs | [Richman et al., 2020](#_ENREF_151); [Weber et al., 2021](#_ENREF_185); [Zhang et al., 2019a](#_ENREF_197); [Liu et al., 2020d](#_ENREF_111); [Labanieh et al., 2022](#_ENREF_93) |
|  |  | Light controllable CAR | [Huang et al., 2020b](#_ENREF_75); [Nguyen et al., 2021](#_ENREF_133) |
|  |  | Ultrasound controllable CAR | [Wu et al., 2021](#_ENREF_189) |
